# Glycerol supports growth of the *Trypanosoma brucei* bloodstream forms in the absence of glucose: Analysis of metabolic adaptations on glycerol-rich conditions

**DOI:** 10.1371/journal.ppat.1007412

**Published:** 2018-11-01

**Authors:** Erika Pineda, Magali Thonnus, Muriel Mazet, Arnaud Mourier, Edern Cahoreau, Hanna Kulyk, Jean-William Dupuy, Marc Biran, Cyril Masante, Stefan Allmann, Loïc Rivière, Brice Rotureau, Jean-Charles Portais, Frédéric Bringaud

**Affiliations:** 1 Laboratoire de Microbiologie Fondamentale et Pathogénicité (MFP), Université de Bordeaux, CNRS UMR-5234, Bordeaux, France; 2 Centre de Résonance Magnétique des Systèmes Biologiques (CRMSB), Université de Bordeaux, CNRS UMR-5536, Bordeaux, France; 3 Institute of Biochemistry and Genetics of the Cell (IBGC) du CNRS, Université de Bordeaux, Bordeaux, France; 4 LISBP, Université de Toulouse, CNRS, INRA, INSA, Toulouse, France; 5 Centre de Génomique Fonctionnelle, Plateforme Protéome, Université de Bordeaux, Bordeaux, France; 6 Trypanosome Transmission Group, Trypanosome Cell Biology Unit, Department of Parasites and Insect Vectors, INSERM U1201, Institut Pasteur, Paris, France; Washington University School of Medicine, UNITED STATES

## Abstract

The bloodstream forms of *Trypanosoma brucei* (BSF), the parasite protist causing sleeping sickness, primarily proliferate in the blood of their mammalian hosts. The skin and adipose tissues were recently identified as additional major sites for parasite development. Glucose was the only carbon source known to be used by bloodstream trypanosomes to feed their central carbon metabolism, however, the metabolic behaviour of extravascular tissue-adapted parasites has not been addressed yet. Since the production of glycerol is an important primary function of adipocytes, we have adapted BSF trypanosomes to a glucose-depleted but glycerol-rich culture medium (CMM_Glyc/GlcNAc) and compared their metabolism and proteome to those of parasites grown in standard glucose-rich conditions (CMM_Glc). BSF were shown to consume 2-folds more oxygen per consumed carbon unit in CMM_Glyc/GlcNAc and were 11.5-times more sensitive to SHAM, a specific inhibitor of the plant-like alternative oxidase (TAO), which is the only mitochondrial terminal oxidase expressed in BSF. This is consistent with (*i*) the absolute requirement of the mitochondrial respiratory activity to convert glycerol into dihydroxyacetone phosphate, as deduced from the updated metabolic scheme and (*ii*) with the 1.8-fold increase of the TAO expression level compared to the presence of glucose. Proton NMR analysis of excreted end products from glycerol and glucose metabolism showed that these two carbon sources are metabolised through the same pathways, although the contributions of the acetate and succinate branches are more important in the presence of glycerol than glucose (10.2% *versus* 3.4% of the excreted end products, respectively). In addition, metabolomic analyses by mass spectrometry showed that, in the absence of glucose, ^13^C-labelled glycerol was incorporated into hexose phosphates through gluconeogenesis. As expected, RNAi-mediated down-regulation of glycerol kinase expression abolished glycerol metabolism and was lethal for BSF grown in CMM_Glyc/GlcNAc. Interestingly, BSF have adapted their metabolism to grow in CMM_Glyc/GlcNAc by concomitantly increasing their rate of glycerol consumption and decreasing that of glucose. However, the glycerol kinase activity was 7.8-fold lower in CMM_Glyc/GlcNAc, as confirmed by both western blotting and proteomic analyses. This suggests that the huge excess in glycerol kinase that is not absolutely required for glycerol metabolism, might be used for another yet undetermined non-essential function in glucose rich-conditions. Altogether, these data demonstrate that BSF trypanosomes are well-adapted to glycerol-rich conditions that could be encountered by the parasite in extravascular niches, such as the skin and adipose tissues.

## Author summary

Until very recently, the bloodstream forms (BSF) of the *Trypanosoma brucei* group species have been considered to propagate exclusively in the mammalian fluids, including the blood, the lymphatic network and the cerebrospinal fluid. All these fluids are rich in glucose, which is widely considered by the scientific community as the only carbon source used by the parasite to feed its central carbon metabolism and its ATP production. Here, we show for the first time that the BSF trypanosomes efficiently grow in glucose-free conditions as long as glycerol is supplied. The raison d'être of this capacity developed by BSF trypanosomes to grow in glycerol-rich conditions regardless of the glucose concentration, including in glucose-free conditions, is not yet understood. However, the recent discovery that trypanosomes colonize and proliferate in the skin and the adipose tissues of their mammalian hosts may provide a rational explanation for the development of a glycerol-based metabolism in BSF. Indeed, the adipocytes composing adipose tissues and also abundantly present in subcutaneous layers excrete large amounts of glycerol produced from the catabolism of glucose and triglycerides. We also show that BSF trypanosomes adapted to glucose-depleted conditions activate gluconeogenesis to produce the essential hexose phosphates from glycerol metabolism. Interestingly, the constitutive expression of the key gluconeogenic enzyme fructose-1,6-bisphosphatase, which is not used for glycolysis, suggests that BSF trypanosomes maintained in the standard glucose-rich medium are pre-adapted to glucose-depleted conditions. This further strengthens the new paradigm that BSF trypanosomes can use glycerol in tissues producing this carbon source, such as the skin the adipose tissues.

## Introduction

*Trypanosoma brucei* is an extracellular protist parasite that causes Human African Trypanosomiasis (HAT) or sleeping sickness, a neglected tropical disease in Sub-Saharan Africa [1]. This parasite undergoes a complex life cycle from the bloodstream of a mammalian host (bloodstream forms—BSF) to the alimentary tract (procyclic form—PCF) and the salivary glands (epimastigote and metacyclic forms) of its blood-feeding insect vector (the tsetse) [2]. There is no vaccine against HAT and the available drugs are difficult to administer and present a number of side effects [3]. Importantly, up to 10% relapses after treatment have been reported, probably due to resurgences of the original infecting strains [4, 5]. In addition, tsetse flies may become infected after feeding on microscopy-negative infected humans or pigs, showing that these apparently aparasitaemic hosts actually host the parasite [6, 7]. Altogether, these observations strongly suggest the existence of extravascular anatomical reservoirs of parasites in the mammalian host that remained unknown until recently. Indeed, this long-lasting question has recently been answered by the description in well-established mouse models that the BSF show a marked tropism to the skin [8, 9], from which transmission to the tsetse vector can occur [9], as well as to adipose tissue [10]. Strikingly, trypanosomes were also detected in the skin of human subjects from a HAT endemic area [9]. Furthermore, within the mouse skin, some parasites were seen in close contact with dermal adipocytes, the major constituent of fat, suggesting that the trypanosome-adipocyte interaction may confer a selective advantage to *T*. *brucei* [8].

In the bloodstream of the mammalian host, the pleomorphic BSF proliferate as long-slender BSF or differentiate into the non-proliferative short-stumpy BSF that are pre-adapted to a further differentiation into PCF in the tsetse midgut [11]. These parasitic forms of *T*. *brucei* share the particularity to possess glycosomes, peroxisome-derived organelles that contain enzymes required for glycolysis. However, PCF and BSF trypanosomes have developed different strategies to produce ATP. Between bloodmeals, the tsetse digestive tract contains negligible amounts of glucose and the fly mostly uses proline as a main carbon source for flight [12–14]. In this context, PCF have developed a proline-based central metabolism and rely on proline metabolism to establish an infection in the fly midgut [15, 16]. Proline is mainly converted by PCF into excreted alanine through part of the tricarboxylic acid (TCA) cycle, with production of ATP by oxidative phosphorylation from the mitochondrial F_O_/F_1_-ATP synthase fed by the proton gradient generated by the respiratory chain [17]. However, the presence of glucose induces a metabolic switch toward glycolysis, accompanied with a down-regulation of proline metabolism [17]. In these glucose-rich conditions, glucose is converted into the excreted end products succinate and acetate, and most ATP molecules are produced by substrate level phosphorylation [18].

**Fig 1:**
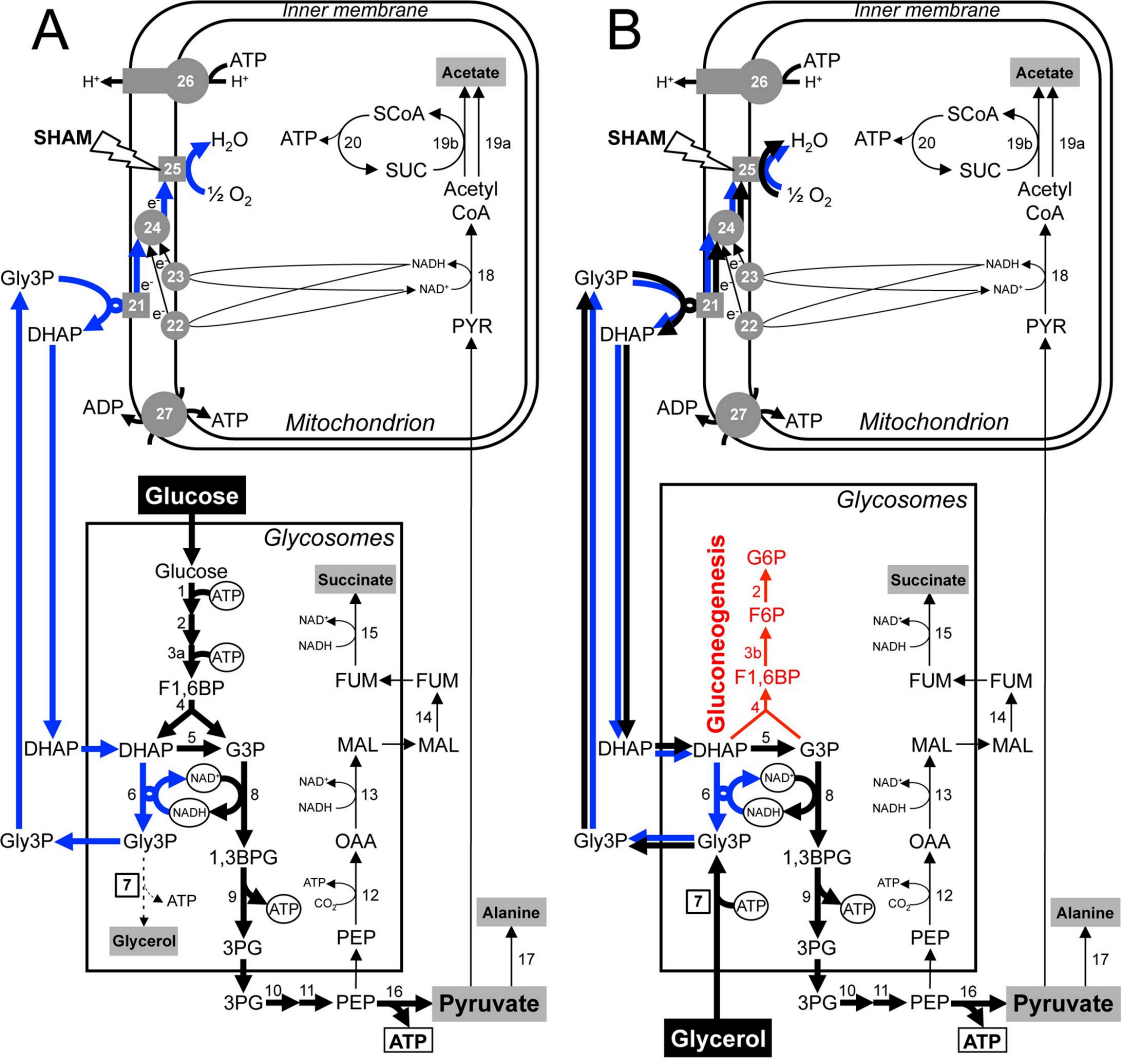
Glucose and glycerol aerobic metabolism of BSF trypanosomes. Panel A shows a schematic representation of the glucose metabolism in BSF trypanosomes grown in glucose-rich medium (CMM_Glc), while panel B shows their glycerol metabolism in the absence of glucose (CMM_Glyc/GlcNAc). The approximately 10-fold higher metabolic flux in glycolysis (down to pyruvate) compared to that of pathways leading to succinate, acetate and alanine is illustrated by a proportional arrow thickness. End products excreted from metabolism of glucose and glycerol are shown in a grey rectangle. In Panel A, the GK step involved in glycerol production from glucose mainly under anaerobiosis is represented by dotted lines. The boxed number corresponds to the enzyme under investigation (GK) and the boxed ATP highlights the enzymatic step providing most of the cellular ATP. The circled glycosomal ATP, NAD^+^ and NADH highlight the main enzymatic steps involved in the maintenance of the glycosomal redox and ATP/ADP balances. The pathway used to maintain the glycosomal redox balance is highlighted by blue arrows, while the same pathway also used for conversion of glycerol-derived Gly3P into DHAP is shown with black arrows in panel B. The target of the SHAM metabolic drug (alternative oxidase) is indicated and the gluconeogenesis from glycerol-derived DHAP and G3P is shown in red. Abbreviations: DHAP, dihydroxyacetone phosphate; e-, electrons; F1,6BP fructose 1,6-bisphosphate; 1,3BPG, 1,3-bisphosphoglycerate; FUM, fumarate; F6P, fructose 6-phosphate; G3P, glyceraldehyde 3-phosphate; G6P, glucose 6-phosphate; Gly3P, glycerol 3-phosphate; MAL, malate; OAA, oxaloacetate; PEP, phosphoenolpyruvate; 3PG, 3-phosphoglycerate; PYR, pyruvate; SCoA, succinyl-CoA; SHAM, salicylhydroxamic acid; SUC, succinate. Indicated enzymes are: 1, hexokinase; 2, glucose-6-phosphate isomerase; 3a, phosphofructokinase; 3b, fructose-1,6-bisphosphatase (FBPase); 4, aldolase; 5, triose-phosphate isomerase; 6, glycerol-3-phosphate dehydrogenase; 7, glycerol kinase (GK); 8, glyceraldehyde-3-phosphate dehydrogenase; 9, phosphoglycerate kinase; 10, phosphoglycerate mutase; 11, enolase; 12, phosphoenolpyruvate carboxykinase; 13, glycosomal malate dehydrogenase; 14, fumarase; 15, NADH-dependent fumarate reductase; 16, pyruvate kinase; 17, L-alanine aminotransferase; 18, pyruvate dehydrogenase complex; 19a, acetyl-CoA thioesterase; 19b, acetate:succinate CoA-transferase; 20, succinyl-CoA synthetase; 21, mitochondrial FAD-dependent glycerol-3-phosphate dehydrogenase; 22, rotenone-sensitive NADH dehydrogenase (complex I of the respiratory chain); 23, rotenone-insensitive NADH dehydrogenase; 24, ubiquinone pool; 25, alternative oxidase (TAO); 26, mitochondrial F_O_/F_1_-ATP synthase; 27, mitochondrial ADP/ATP exchanger.

In contrast, it is widely accepted that slender BSF exclusively rely upon glycolysis as an energy source. In this context, the down-regulated TCA cycle is now poorly or not active and the respiratory chain does not produce ATP anymore [19]. It was generally accepted that the proliferative slender BSF grown under aerobiosis convert glucose exclusively into pyruvate [20–22]. However, it has recently been demonstrated that BSF actually also convert ~15% of the consumed glucose into excreted acetate, alanine and succinate, with a rate of acetate excretion from glucose breakdown comparable to that in PCF (Fig 1A) [23]. More importantly, key enzymes involved in the production of these three "minor" glycolytic end products are essential for BSF viability, *i*.*e*. the alanine aminotransferase [24], the pyruvate dehydrogenase complex [23] and the phosphoenolpyruvate carboxykinase [25]. This highlights the fact that the central metabolism of BSF trypanosomes is probably much more elaborate and flexible than initially considered.

Here, we show for the first time that BSF can readily and efficiently adapt their central metabolism to a glycerol-rich medium in the absence of glucose, not only for survival but also to sustain a long-term proliferation. These data further illustrate the adaptive capacity of African trypanosomes and pave the way to study the metabolism of the mammalian forms of the parasite in extravascular compartments such as adipose tissues and especially in the skin, which is also crucial for parasite transmission and where glycerol may be an alternative carbon source.

## Results

### Glycerol can replace glucose for BSF trypanosomes to grow

**Fig 2:**
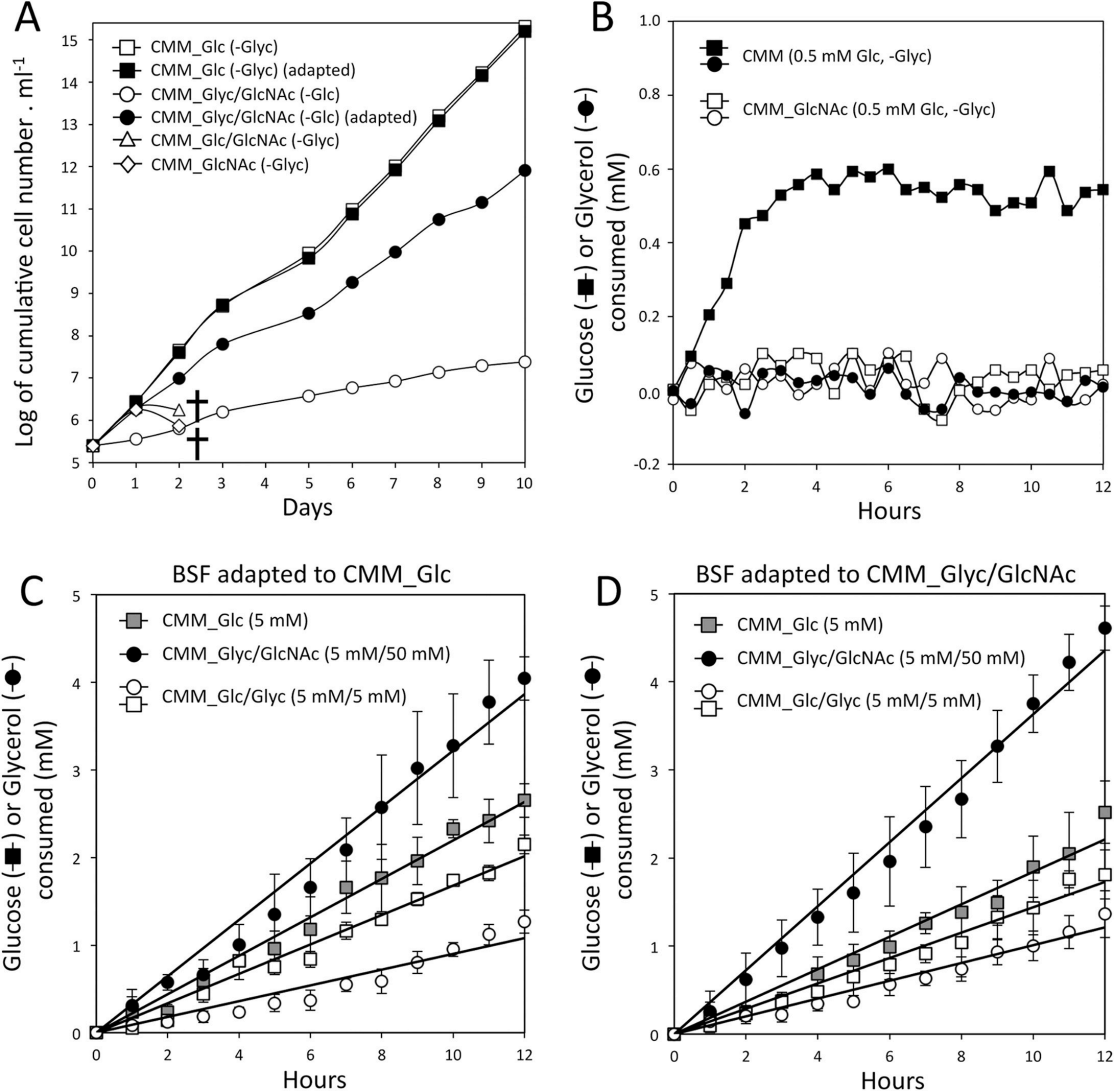
Glycerol supports BSF growth in the absence of glucose. Panel A shows growth curves of the 427 90–13 BSF parental strain in Creek's minimal medium (CMM) containing 10 mM glucose (CMM_Glc), 10 mM Glucose and 50 mM GlcNAc (CMM_Glc/GlcNAc), 10 mM glycerol and 50 mM GlcNAc (CMM_Glyc/GlcNAc), or only 50 mM GlcNAc without glucose and glycerol (CMM_GlcNAc). Filled squares and circles mean that the cells have been adapted during 3 months in CMM_Glc or CMM_Glyc/GlcNAc, respectively, before starting the growth monitoring. The crosses mean that all the cells were dead. Panel B shows glucose and glycerol consumption over time by the 427 90–13 BSF strain incubated in CMM and CMM_GlcNAc containing 0.5 mM glucose and no glycerol. Panels C and D show the same experiment as in panel B with cells incubated in CMM_Glc, CMM_Glyc/GlcNAc or CMM_Glc/Glyc (CMM containing equimolar amounts of glucose and glycerol) containing 5 mM glucose and/or glycerol (error bars indicate means ± SD of 3 biological replicates). Before the experiment, BSF have been grown for at least one month in CMM_Glc (panel C) or CMM_Glyc/GlcNAc (panel D).

To identify carbon sources alternative to glucose, we developed a glucose-depleted medium based on the Creek's minimal medium (CMM) [26] containing 50 mM N-acetyl glucosamine (GlcNAc) to inhibit the uptake of the FCS-derived glucose (0.5 mM final concentration in the minimal medium). GlcNAc is a competitive inhibitor of the *T*. *brucei* glucose transporters, as demonstrated by its lethal effect on monomorphic *T*. *brucei* 427 90–13 BSF in either glucose-rich CMM (CMM_Glc/GlcNAc, 10 mM glucose) or glucose-depleted CMM (CMM_GlcNAc, 0.5 mM glucose) (Fig 2A). The glucose-transport inhibition effect of GlcNAc was confirmed by the abolition of glucose consumption by BSF trypanosomes in the presence of a 100-fold excess of GlcNAc (50 mM GlcNAc) compared to glucose (0.5 mM) (Fig 2B). Strikingly, the 427 90–13 BSF cell line was able to sustain a long-term proliferation in the glucose-depleted CMM containing GlcNAc after addition of 10 mM glycerol (CMM_Glyc/GlcNAc conditions). Transfer of the CMM_Glc-adapted BSF in the CMM_Glyc/GlcNAc medium strongly affected growth of the parasite, which was partially restored after at least one month of adaptation in the CMM_Glyc/GlcNAc conditions, with a doubling time only ~30% higher than in glucose-rich conditions (CMM_Glc) (11 ± 1.6 h *versus* 7.3 ± 0.4 h) (Fig 2A).

### BSF metabolise glycerol as efficiently as glucose

To investigate the capability of BSF to use glycerol, we measured the rates of glycerol consumption in cells pre-adapted or not to CMM_Glyc/GlcNAc and compared these data to the rates of glucose consumption. Cells were adapted for one month in either CMM_Glyc/GlcNAc or CMM_Glc and then were transferred to fresh CMM medium containing either glycerol/GlcNAc, glucose or glycerol/glucose to measure the consumption of the carbon sources. The rates of glucose and glycerol consumption were determined by enzymatic assay of the carbon sources remaining in the CMM medium. Since glucose contains 6 carbons and glycerol only 3, data were expressed as carbon molecules equivalent (mol.C) for comparison purpose. Cells adapted to CMM_Glyc/GlcNAc consumed glycerol at the rate of 57.9 ± 1.8 μmol.C per h per 10^8^ cell (Fig 2C and Table 1). This was lower than the rate of glucose consumption by cells adapted to CMM_Glc medium (69.0 ± 3.6 μmol.C per h per 10^8^ cell), which can explain the lower growth rate on glycerol compared to glucose. It is noteworthy that after adaptation to CMM_Glyc/GlcNAc conditions, the rate of glycerol consumption increased by 9.9%, while the rate of glucose consumption is reduced by 13%, respectively, compared to cells adapted to CMM_Glc (Table 1).

**Table 1:** Rate of glucose and glycerol consumption in BSF adapted to CMM_Glc or CMM_Glyc/GlcNAc.

	Consumption rate (μmol.h^-1^.10^8^ cells^-1^)							
	Adapted to CMM_Glc^*a*^				Adapted to CMM_Glyc/GlcNAc^*a*^			
Incubation condition^*b*^	Glucose	Glycerol	mol.C^*c*^	Total rate (C.mol)	Glucose	Glycerol	mol.C^*c*^	Total rate (C.mol)
CMM_Glc	11.5 ± 0.6		69.0 ± 3.6	**69.0 ± 3.6**^*d*^	10.0 ± 1.1		60.0 ± 6.6	60.0 ± 6.6
CMM_Glyc/GlcNAc		17.4 ± 1.9	52.2 ± 6.0	52.2 ± 6.0		19.3 ± 0.6	57.9 ± 1.8	**57.9 ± 1.8**^*d*^
CMM_Glc/Glyc	9.0 ± 0.3		54.0 ± 1.8	69.6 ± 3.6	7.7 ± 0.8		46.2 ± 5.4	62.4 ± 7.2
		5.2 ± 0.6	15.6 ± 0.9			5.4 ± 0.7	16.2 ± 2.4	

^*a*^ Growth conditions; the cells have been grown in CMM_Glc or CMM_Glyc/GlcNAc before incubation in CMM_Glc, CMM_Glyc/GlcNAc or CMM_Glc/Glyc for the 12 h measurement of the consumed carbon sources (see Fig 2)

^*b*^ Medium used for the 12 h incubation

^*c*^ Number of carbon molecules consumed, considering that glucose and glycerol are composed of 6 and 3 carbons, respectively

^*d*^ Rate of glucose and glycerol consumption in cells adapted to CMM_Glc and CMM_Glyc/GlcNAc, respectively, expressed as number of carbon molecules consumed

**Fig 3:**
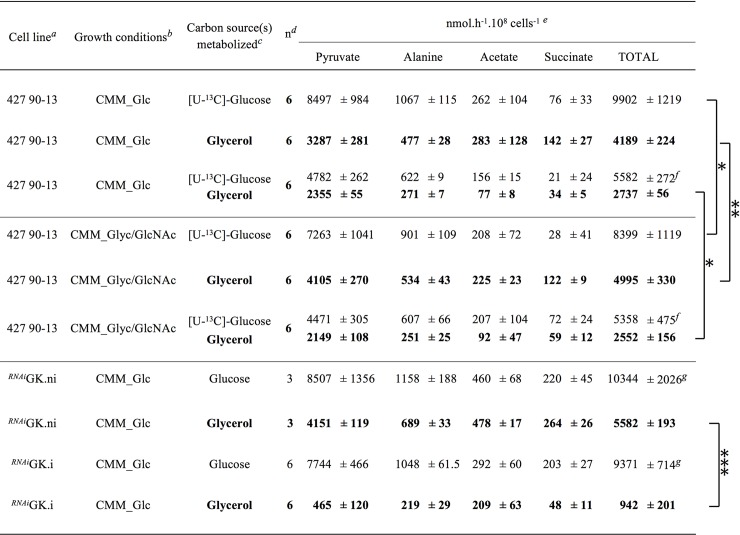
Excreted end products of glucose and glycerol metabolism by BSF parental and mutant cell lines. ^*a*^.i: RNAi cell line induced during 5 days by addition of tetracycline; .ni: non-induced RNAi cell line. ^*b*^ The cells have been grown in CMM_Glc or CMM_Glyc/GlcNAc before incubation in PBS containing one or two carbon sources. ^*c*^ Carbon source(s) added in the incubation medium (PBS). ^*d*^ Number of biological replicates. ^*e*^ The amount of glycerol excreted from glucose metabolism is not estimated because of overlapping resonances between glucose and glycerol. ^*f*,*g*^ Data not significantly different with p-value ≤0.05. *, **, *** Data significantly different with p-value ≤0.05, ≤0.01 and ≤0.001, respectively.

We compared the release of end products by BSF incubated in PBS containing glucose and/or glycerol using the ^1^H-NMR profiling approach previously developed [27]. As previously observed, BSF cultivated in CMM_Glc mainly converted glucose into pyruvate (85.8% of the excreted end products) and alanine (10.8%), with lower amounts of acetate (2.6%) and succinate (0.8%) (Fig 3) [23, 25]. It is noteworthy that glycerol possibly produced from glucose cannot be quantified using our NMR approach due to overlapping resonances between glucose and glycerol. Glycerol was also converted into these four excreted end products, with higher ratio of acetate (6.8%) and succinate (3.4%), while the alanine production remained in the same range (11.4%) and the pyruvate proportion was decreased (78.5%) (Fig 3). This suggests that glycerol was indeed catabolised through the same pathways as glucose, yet with slight differences in the flow distribution within the different branches of the network. The rate of all end product excretion from glycerol and glucose consumption in cells adapted to CMM_Glyc/GlcNAc were increased by 16.1% and reduced by 15.2%, respectively, as compared to cells adapted to CMM_Glc, which was consistent with the rates of carbon source consumption described above (Table 1 and Fig 3).

### Glycerol catabolism occurs in the presence of glucose

When BSF were grown in the presence of equal amounts of glucose and glycerol (CMM_Glc/Glyc, 5 mM each), both carbon sources were consumed, although the parasites showed a preference for glucose. Indeed, cells adapted to CMM_Glc consumed glycerol at a rate 3.3-fold lower in CMM_Glc/Glyc compared to CMM_Glyc/GlcNAc, while the rate of glucose consumption was only 1.3-fold reduced in CMM_Glc/Glyc compared to CMM_Glc (Fig 2C and Table 1). These ratios were equivalent for cells adapted to CMM_Glyc/GlcNAc (3.5 and 1.3, respectively) (Fig 2D and Table 1). Considering that two molecules of glycerol (3 carbons) are the equivalent to one molecule of glucose (6 carbons), this implies that glucose contributes ~3.5-times more than glycerol to the central carbon metabolism at equimolar concentration (54 *versus* 15.6 μmol.C per h and per 10^8^ cells, see Table 1). It is noteworthy that the overall carbon consumption was maintained in CMM_Glc and CMM_Glc/Glyc in both cells adapted to CMM_Glc (69 and 69.6 μmol.C per h and per 10^8^ cells, respectively) and cells adapted to CMM_Glyc/GlcNAc (60 and 62.4 μmol.C per h and per 10^8^ cells, respectively).

To confirm these data, quantitative analyses of excreted end products from glucose and/or glycerol metabolism were achieved by using a metabolite profiling assay based on the ability of ^1^H-NMR spectrometry to distinguish ^13^C-enriched molecules from ^12^C molecules. To discriminate the metabolic origin of end products, cells were incubated in PBS with equal amounts (4 mM) of uniformly [^13^C]-enriched glucose ([U-^13^C]-glucose) plus glycerol. In these experiments, labelled end products and unlabelled end products were derived from glucose and glycerol, respectively [27, 28]. Cells adapted to CMM_Glc and CMM_Glyc/GlcNAc excreted 2.0- and 2.1-times more end products from the catabolism of glucose than from glycerol (Fig 3), which means that glucose contributed ~2-times more to the carbon supply to central metabolism. This was consistent with the higher contribution of glucose observed in rich medium CMM_Glc/Glyc (Table 1). These data also showed that the BSF preference for glucose remained equivalent after adaption to CMM_Glyc/GlcNAc (Fig 3).

### Glycerol metabolism strictly relies on oxygen

**Fig 4:**
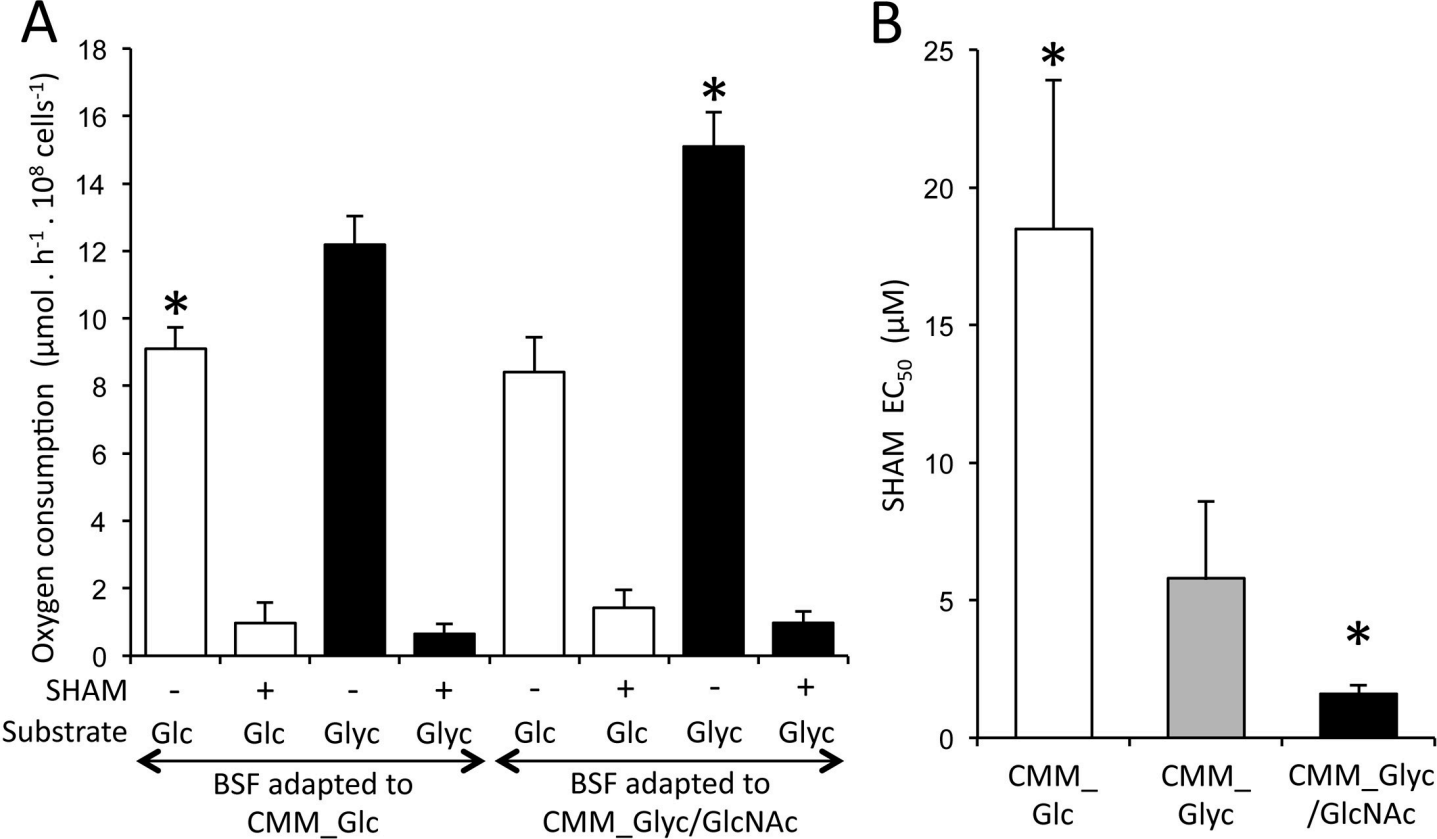
Comparison of oxygen consumption and sensitivity to metabolic inhibitors. Panel A shows oxygen consumption of parental BSF adapted to CMM_Glc or CMM_Glyc/GlcNAc growth conditions and resuspended in fresh CMM containing glucose (Glc, white) or glycerol/GlcNAc (Glyc, black) in the presence (+) or not (-) of 4 mM SHAM (error bars indicate means ± SD of 9 experiments, including 3 biological replicates). The SHAM EC_50_ values (μM) in BSF adapted to CMM_Glc (white), CMM_Glyc (grey) or CMM_Glyc/GlcNAc (black) are shown in panel B. Error bars indicate means ± SD of 3 biological replicates. The asterisks indicate data relevant for BSF adapted to CMM_Glc (white) and CMM_Glyc/GlcNAc (Black).

Although the growth rate was reduced in BSF adapted to CMM_Glyc/GlcNAc, the rate of endogenous oxygen consumption was higher in the presence of glycerol compared to glucose. Indeed, cells adapted to CMM_Glyc/GlcNAc consumed 1.64-times more oxygen from glycerol compared to oxygen consumption from glucose of cells adapted to CMM_Glc (Fig 3A, asterisk). These data were consistent with the glucose and glycerol metabolic pathways schematized in Fig 1A and 1B, respectively. Indeed, only one molecule of oxygen (2 x ½ O_2_) is required to regenerate, through the glycerol phosphate shuttle (steps 6, 21, 24 and 25; blue pathway in Fig 1), two molecules of NADH produced upon glucose conversion into two molecules of 1,3-bisphosphoglycerate (step 8). In contrast, two oxygen molecules are required to metabolise two molecules of glycerol (equivalent to one molecule of glucose). This additional oxygen molecule is necessary to convert the glycerol-derived glycerol 3-phosphate into dihydroxyacetone phosphate through the glycerol phosphate shuttle (steps 7, 21, 24 and 25, black pathway in Fig 1B). According to our current knowledge on BSF metabolism, respiration strictly depends on the alternative oxidase activity (TAO, step 25), which is sensitive to salicylhydroxamic acid (SHAM) [29]. Indeed, respiration of BSF incubated in CMM_Glc and CMM_Glyc/GlcNAc was almost abolished by the addition of 4 mM SHAM (Fig 4A). Moreover, the BSF respiration in the presence of glucose or glycerol was affected neither by addition of 2 μM CCCP, an uncoupling agent dissipating the mitochondrial proton gradient, nor by the addition of 4 mM oligomycin, a specific inhibitor of the F_O_/F_1_-ATP synthase that generates the proton gradient in BSF [30, 31]. This confirmed that respiration is not linked to the proton gradient and is primarily driven by TAO, regardless the carbon source used between glucose or glycerol.

Considering that oxygen is absolutely required for DHAP production from glycerol (see Fig 1B), although glucose can be catabolised in the absence of oxygen to produce glycerol (see Fig 1A) [32], we reasoned that BSF sensitivity to SHAM-induced TAO inhibition could depend on the carbon source metabolised by BSF. As expected, the 427 90–13 BSF cell line maintained in CMM_Glyc/GlcNAc was 11.5-times more sensitive to SHAM compared to the same cell line grown in CMM_Glc (EC_50_ of 1.6 ± 0.3 μM *versus* 18.5 ± 5.4 μM, respectively) (Fig 4B). An intermediate sensitivity to SHAM was observed in cells grown in CMM_Glyc (EC_50_ of 5.8 ± 2.8 μM), certainly due to the consumption of the residual 0.5 mM glucose present in the medium (Fig 4B).

It is noteworthy that oxygen consumption from glycerol and glucose metabolism of cells adapted to CMM_Glyc/GlcNAc increased by 18% and was reduced by 7.6%, respectively, compared to cells adapted to CMM_Glc, which was consistent with the rate of glucose and glycerol consumption in of cells adapted to these two growth conditions (Table 1).

### Glycerol feeds gluconeogenesis in CMM_Glyc/GlcNAc

**Fig 5:**
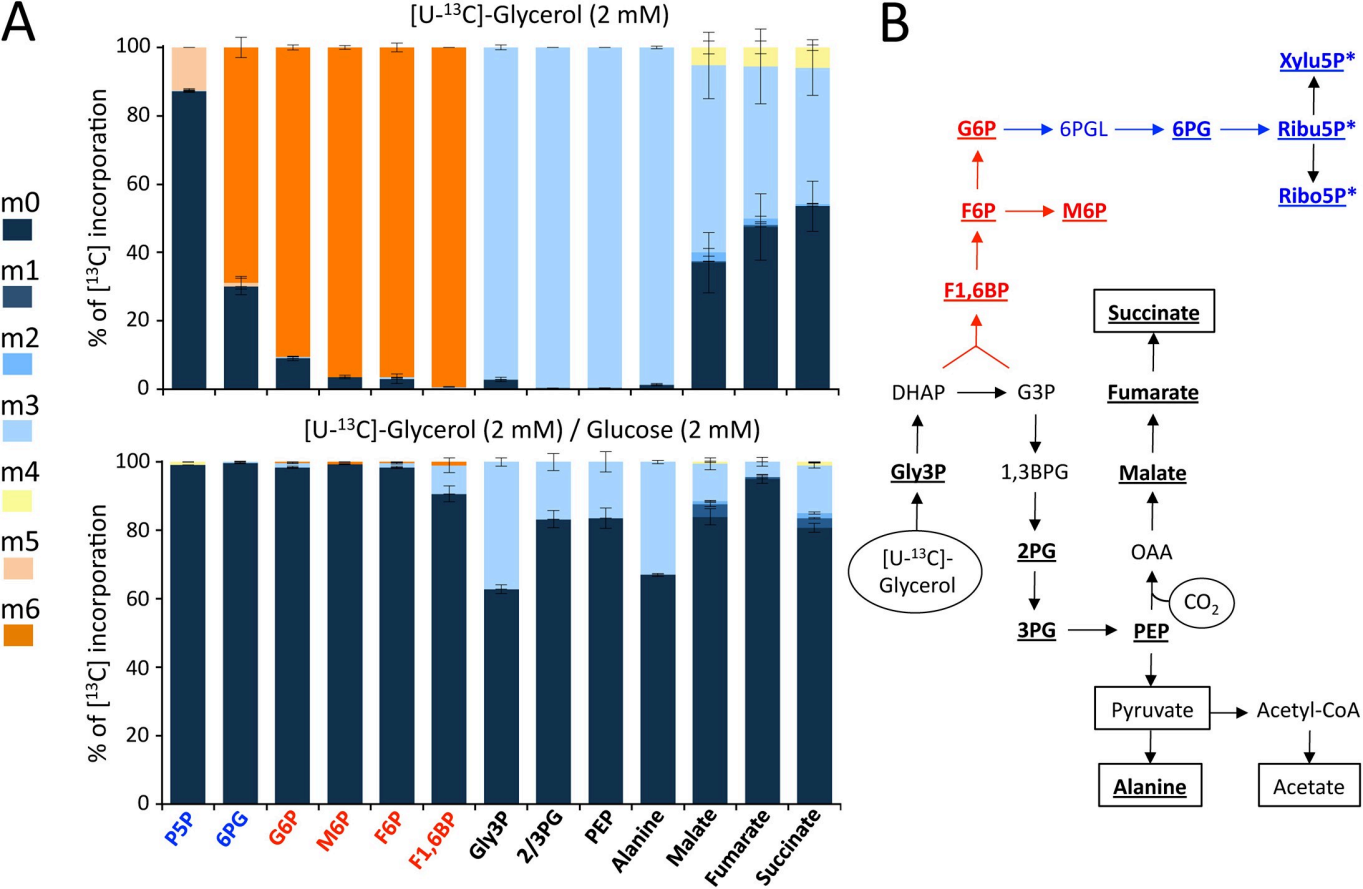
Incorporation of [U-^13^C]-glycerol into BSF intracellular metabolites. The 427 90–13 BSF parental cell line was incubated for 1 h in PBS containing 2 mM [U-^13^C]-glycerol alone or in the presence of 2 mM glucose (Panel A). The figure shows the ^13^C-enrichment of key metabolites of the intermediate metabolism at 0 to 6 carbon positions (m0 to m6, colour code indicated, "mx" denotes metabolites in which x of the atoms are ^13^C instead of ^12^C) with ^13^C expressed as percentage of all corresponding molecules (MID; Mass Isotopomer Distribution). Error bars indicate means ± SD of 3 biological replicates. The metabolic scheme in panel B represents the enzymatic reactions leading to production of the analysed metabolites from glycerol metabolism (bold faced and underlined ones). Gluconeogenesis and mannose 5-phosphate production are shown in red and part of the pentose phosphate pathway is in blue. The carbon sources incorporated in the metabolism network ([U-^13^C]-glycerol and CO_2_) are circled and the excreted end products are boxed. For abbreviations see Fig 1, other abbreviations are: Ala, alanine; M6P, mannose 6-phosphate; 2/3PG, 2- or 3-phosphoglycerate (these two metabolites are undistinguished by IC-MS/MS); 6PG, 6-phosphogluconate; 6PGL, 6-phosphogluconolactone; P5P, pentose 5P including ribose 5-phosphate (Ribo5P), ribulose 5-phosphate (Ribu5P) and xylulose 5-phosphate (Xylu5P), which are undistinguished by IC-MS/MS.

To further dissect the metabolic fluxes involved in BSF adaptation to glycerol in the absence of glucose, we reasoned that one major pathway could be gluconeogenesis, a glycosomal function that remained unexplored in BSF trypanosomes so far. In the CMM_Glyc/GlcNAc context, gluconeogenesis should be activated in order to produce the glucose 6-phosphate (G6P) precursor required for several pathways that are essential for the cells, such as the production of the GPI anchors used for VSG biosynthesis. To investigate the unexpected and unexplored gluconeogenic metabolic pathway in the 427 90–13 BSF strain, we determined by mass spectrometry the incorporation of ^13^C atoms from uniformly [^13^C]-enriched glycerol ([U-^13^C]-glycerol) into glycolytic intermediates, as well as other key metabolites of the intermediary metabolism. Cells were incubated 1 h in the presence of 2 mM [U-^13^C]-glycerol and incorporation of ^13^C into metabolites was quantified by IC-MS/MS (Fig 5A, top panel). Glycerol rapidly fed the central carbon metabolism as deduced from >98% of fully labelled triose phosphates and alanine. However, only 50–66% of the dicarboxylic acid molecules, *i*.*e*. malate, fumarate and succinate, contained at least one ^13^C-carbon, which was consistent with the relatively low contribution of the succinate production pathway compared to the pyruvate production (see Fig 3). The high proportion of 3-carbon labelled dicarboxylic acids (30–50%), instead of 4-carbon (3–5%), was due to the carboxylation of phosphoenolpyruvate into oxaloacetate (Figs 1B and 5B). Glycerol was also converted into hexose phosphates, with 100% (fructose 1,6-bisphosphate) to 90% (G6P) of them being fully ^13^C-enriched, which highlighted the relatively high flux through the gluconeogenic pathway. Glycerol-derived G6P fed the pentose phosphate pathway with 70% and 12.5% of 6-phosphogluconate and pentose 5-phosphate molecules being fully ^13^C-enriched, respectively.

In the presence of equimolar amounts of glucose, the incorporation of label from [U-^13^C]-glycerol into hexose phosphates and intermediates of the pentose phosphate pathway was reduced to background levels (Fig 5A, lower panel). 16% to 37% of the triose-phosphate and alanine molecules were fully labelled, which suggests that in these conditions, glucose contributed ~4-times more than glycerol to central metabolism. This was consistent with the analyses of glucose and glycerol consumption (Table 1) and metabolism (Fig 3) in the same incubation conditions. Altogether, these data showed that BSF trypanosomes have developed the metabolic capacity to produce G6P through gluconeogenesis in the absence of glucose.

### BSF proteome adaptations to a glycerol-based metabolism

To further study the metabolic modifications occurring in BSF trypanosomes adapted to glycerol conditions, the proteome of cells grown in CMM_Glyc/GlcNAc was compared to that of cells grown in CMM_Glc. Among the 2,081 proteins identified by at least 3 different peptides, a total of 1,052 proteins were retained for further analyses after having successfully passed the ANOVA statistical test (p-value ≤0.05). In total, only 29 proteins and isoforms were up- or down-regulated more than 2-fold (2.7% of the proteome) and 109 proteins with a 1.5-fold threshold (10.4% of the proteome), suggesting that BSF are quite well pre-adapted to live and proliferate in a glycerol-rich and glucose-depleted environment (S1 and S2 Tables).

**Fig 6:**
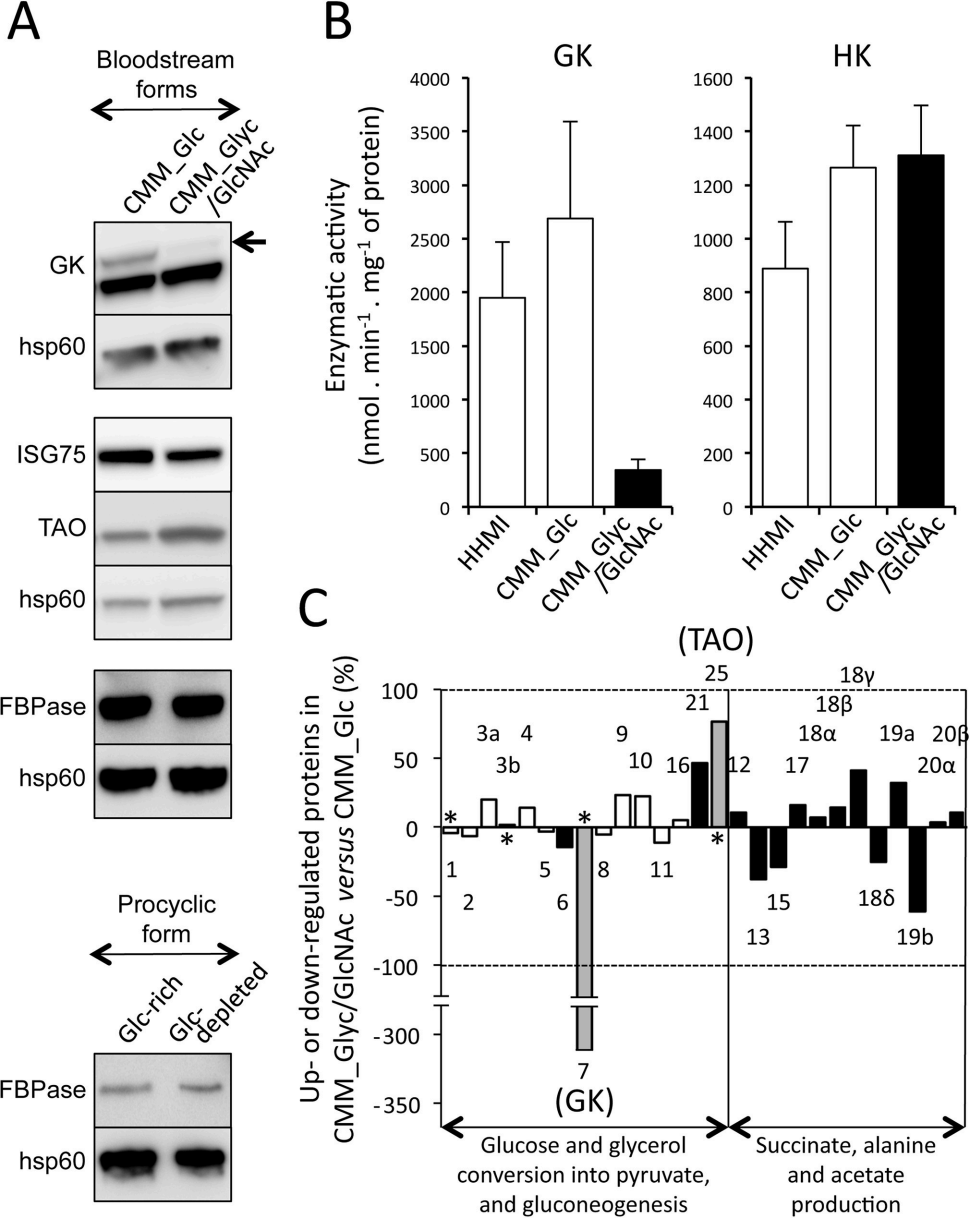
Modulation of the glycerol and glucose metabolic pathways in BSF grown in CMM_Glyc/GlcNAc. The expression of GK, TAO, ISG75 and FBPase in BSF grown in CMM_Glc and CMM_Glyc/GlcNAc, as well as FBPase in procyclic trypanosomes grown glucose-rich and glucose-depleted conditions was analysed by western blotting using the anti-hsp60 immune serum as control (Panel A). The arrow indicates a 55 kDa band corresponding to GK (calculated Mr: 56.4 kDa), above a non-specific signal produced by the anti-GK immune serum. To compare expression of FBPase/hsp60 the analysis of the BSF and PCF samples was performed on the same gel. Panel B shows the GK and HK enzymatic activities in the three growth conditions (error bars indicate means ± SD of 3 biological replicates). The proteomic data described in S3 Table is presented in Panel C by expressing the percentage of up- or down-regulation of the indicated enzymes in cells grown in CMM_Glyc/GlcNAc as compared to cells grown in CMM_Glc. The dashed line corresponds to two-fold up- or down-regulations. The numbers indicate the enzymatic steps described in Fig 1. The subunits of the pyruvate dehydrogenase complex and of the succinyl-CoA synthetase are annotated 18α-18δ and 20α-20β, respectively. Up- and down-expression of TAO and GK, respectively, are represented by grey bars, and that of glycolytic enzymes are shown with white bars. The asterisks mean that the proteome data have been confirmed by western blotting and/or enzymatic activity assay.

Glycerol kinase (GK, EC 2.7.1.30, step 7 in Fig 1), which is encoded by 5 tandemly arranged genes (Tb927.9.12550, Tb927.9.12570, Tb927.9.12590, Tb927.9.12610, Tb927.9.12630) was the most down-regulated protein in CMM_Glyc/GlcNAc (4-fold). This surprising observation was confirmed by western blot analysis (Fig 6A). In addition, the GK activity was 7.8-fold reduced after cell adaptation to CMM_Glyc/GlcNAc, while the hexokinase activity was not affected, as confirmed by the proteome analysis (Fig 6B and 6C, S3 Table). This demonstrated the expression of a large excess of GK in CMM_Glc-adapted BSF, since the 7.8-fold reduction of the GK activity did not induce a reduction of the rate of glycerol consumption. Actually, the rate of glycerol consumption was increased by 11%, although one would have expected the opposite (see Table 1).

Interestingly, the expression of the 75 kDa invariant surface glycoprotein isoforms (ISG75) [33] were 3- to 4-fold down-regulated after cell adaptation in CMM_Glyc/GlcNAc. ISG75 is implicated in the uptake of suramin, the first-line trypanocidal compound against *T*. *b*. *rhodesiense*, indicating that ISGs may act as receptors, although their ligand(s) remain to be identified [34]. ISG75 down-regulation in cells grown in CMM_Glyc/GlcNAc, which was confirmed by western blotting (Fig 6A), is therefore another piece of the puzzle to understand its enigmatic function.

Zooming on enzymes involved in glucose and glycerol metabolisms showed that the expression of all glycolytic enzymes was marginally or not affected by the cell growth in glycerol conditions (ranging from 10% down-regulation to 25% up-regulation) (Fig 6C and S3 Table). Interestingly, the expression of the gluconeogenic enzyme fructose-1,6-bisphosphatase (FBPase, EC 2.7.1.40, step 3b) was unchanged and the glycolytic enzyme performing the reverse reaction, *i*.*e*. phosphofructokinase (PFK, EC 2.7.1.11, step 3a) was moderately up-regulated (20%) in CMM_Glyc/GlcNAc-cultured cells. These data confirmed by western blotting (Fig 6A) indicated that BSF grown upon glucose-rich conditions maintained the ability to perform gluconeogenesis from glycerol, which favours the hypothesis that parasites could face *in vivo* conditions requiring gluconeogenesis. The most up-regulated enzymes involved in both glucose and glycerol metabolism were two mitochondrial enzymes involved in the oxidation of glycosomal NADH, *i*.*e*. the mitochondrial FAD-dependent glycerol-3-phosphate dehydrogenase (FAD-GPDH, EC 1.1.5.3, step 21) and the alternative oxidase (TAO, EC 1.10.3, step 25), that were 1.5- and 1.8-fold up-regulated, respectively (Fig 6C and S3 Table). Up-regulation of the TAO was confirmed by western blotting analysis (Fig 6A), which was in agreement with the increased oxygen consumption from glycerol in cell adapted to CMM_Glyc/GlcNAc as compared to cells grown in glucose-rich conditions (Fig 4A).

### Glycerol kinase is essential for BSF grown in CMM_Glyc/GlcNAc

**Fig 7:**
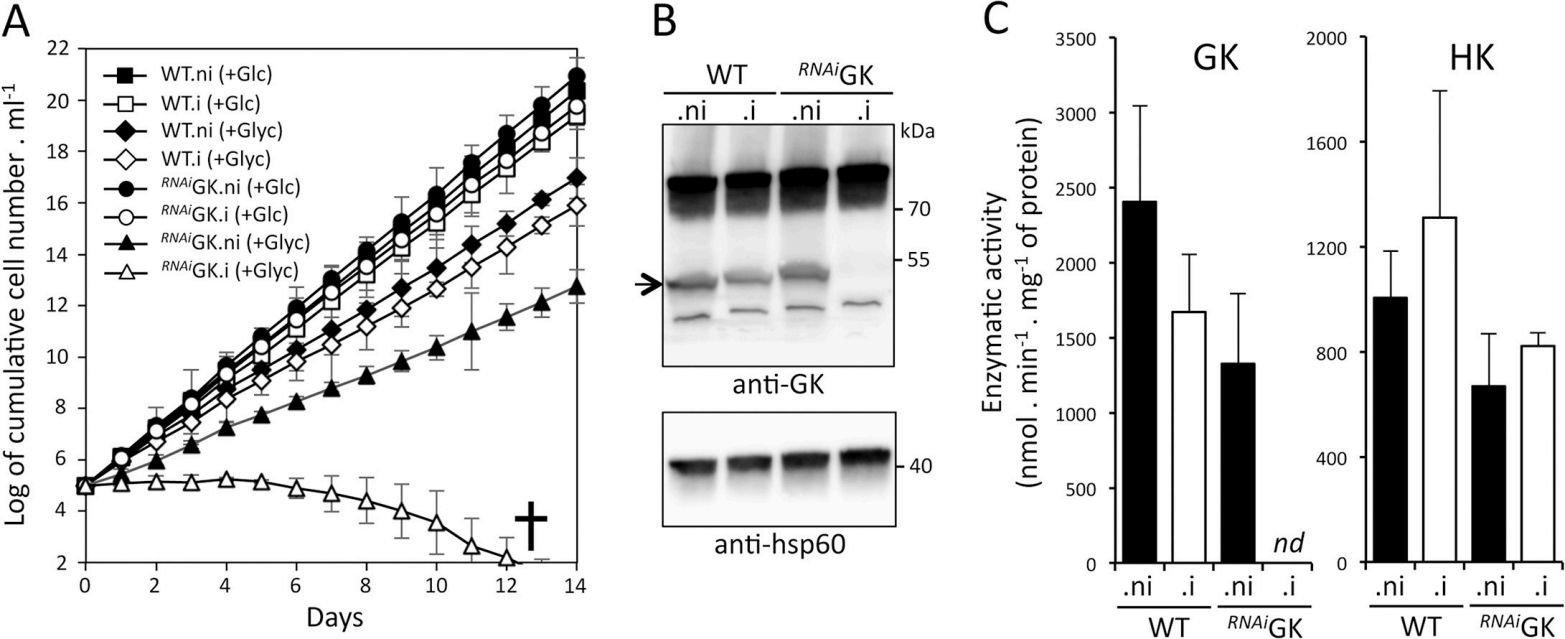
Role of glycerol kinase in glycerol metabolism. Panel A shows growth curves of the parental 427 90–13 BSF (WT) and ^*RNAi*^GK cell lines incubated in CMM_Glc (Glc) or CMM_Glyc/GlcNAc (Glyc), in the presence (.i) or not (.ni) of tetracycline. The western blot controls with the anti-GK and anti-hsp60 (heat shock protein 60) immune sera are shown in the panel B and the GK and HK enzymatic activity of WT cells, as well as un-induced or 5-day induced ^*RNAi*^GK cells are shown in panel C. The anti-GK immune serum reveals non-specific bands in addition to GK, that is indicated by an arrow. *nd* means not detected and error bars in panels A and C indicate the means ± SD of 3 biological replicates.

To further investigate the importance of the *GK* genes, their expression was down-regulated by RNAi in the 427 90–13 BSF strain. The proliferation of the parental strain and that of the ^*RNAi*^GK cell line incubated in CMM_Glc were only moderately affected by the tetracycline-induced expression of GK double-stranded RNAs (^*RNAi*^GK.i). This was consistent with the relative low amounts of glycerol produced from glucose metabolism in the presence of oxygen in the parental cell line [32]. In contrast, the ^*RNAi*^GK.i cell line incubated in CMM_Glyc/GlcNAc died after 12 days of induction (Fig 7A). The GK expression in this ^*RNAi*^GK.i cell line was not detectable any more, neither by western blotting (Fig 7B) nor by enzymatic assay (Fig 7C). The direct involvement of the GK in the glycerol metabolism was further quantified by ^1^H-NMR spectrometry analysis of end products excreted from glycerol metabolism. As expected, the rates of pyruvate, alanine, acetate and succinate excretion from glycerol metabolism were 5.9-fold reduced upon tetracycline-induction of the ^*RNAi*^GK cell line as compared to non-induced cells, while glucose metabolism was not affected (Fig 4 and S1 Fig). It is noteworthy, that glycerol metabolism was not abolished in the ^*RNAi*^GK.i cell line, although both the GK protein and GK activity were not detectable (S1 Fig). These data were consistent with BSF expressing a large excess of GK, far above the minimal amounts required to maintain the high level of glycerol metabolic flux. Nevertheless, this metabolic flux decrease was sufficient to abolish BSF growth in CMM_Glyc/GlcNAc.

## Discussion

*T*. *brucei* BSF are currently considered to strictly depend on glycolysis for their growth and development in their mammalian hosts. Indeed, it is widely accepted that the glucose present in mammalian fluids at relatively high and constant concentrations (5 mM) is the only carbon source used by the parasites for ATP production. It has also previously been reported that BSF can convert glycerol into pyruvate [35–39], however, the relatively low abundance of glycerol in the bloodstream (50–100 μM) [40, 41] and the parasite preference for glucose over glycerol (glucose contributes 4-times more than glycerol to the central metabolism than glycerol when present at equimolar amounts), implies that glucose is indeed the main source of ATP for BSF trypanosomes in mammalian fluids. However, the recent descriptions of trypanosomes residing in the skin and adipose tissues raise new fundamental questions about the metabolism of these extravascular trypanosomes. Here, we report for the first time that BSF are able not only to survive but to establish and maintain a long-term proliferation in a minimum medium containing glycerol instead of glucose, which illustrates a greater metabolic flexibility than appreciated so far.

The comparison of glucose and glycerol metabolisms by BSF trypanosomes, adapted or not to glycerol conditions, reveals two main metabolic differences. First, parasites consume two times more oxygen per carbon unit consumed when glycerol is the carbon source used. This is consistent with the currently accepted metabolic scheme, since the glycerol phosphate redox shuttle to oxygen has to be performed twice for each glycerol molecule consumed in order to maintain the redox balance within the glycosomes (Fig 1B): to first produce dihydroxyacetone phosphate from the glycerol-derived glycerol 3-phosphate (step 21) and then a second time for the re-oxidation of the glycosomal NADH produced by the glyceraldehyde-3-phosphate dehydrogenase reaction by the glycerol-3-phosphate dehydrogenase (steps 8, 6 and 21). In contrast, only a single cycle is required for glucose catabolism to re-oxidize the glycosomal NADH produced by the glyceraldehyde-3-phosphate dehydrogenase reaction (Fig 1A). This also implies that glycerol metabolism is strictly dependent on oxygen, while glycolysis can occur in anaerobiosis, yet with a reduced ATP production rate that does not allow cells to grow [21]. This is illustrated by the 11.5-times higher sensitivity to SHAM, a TAO specific inhibitor, of BSF grown in CMM_Glyc/GlcNAc compared to CMM_Glc (Fig 4B). Consequently, TAO specific inhibitors such as derivatives of the lead compound ascofuranone developed for the treatment of sleeping sickness are predicted to be particularly efficient on BSF residing in glycerol-rich environments [29]. The second metabolic difference is the utilisation of gluconeogenesis to produce hexose phosphates in glycerol-rich conditions. The activation of this pathway in the absence of glucose is expected because of the absolute requirement of G6P to feed some essential pathways, such as the production of GPI anchors required for the biosynthesis of the BSF coat composed of a variable surface glycoprotein. Interestingly, the constant expression level of FBPase, the key gluconeogenic enzyme that is yet not used when cells rely on glycolysis, regardless the growth conditions of BSF (CMM_Glyc/GlcNAc *versus* CMM_Glc) and of PCF (glucose-depleted *versus* glucose-rich) (see Fig 6A), suggests that trypanosomes could maintain their gluconeogenic capacity in standard glucose-rich conditions possibly to be prepared for future environments poor or depleted in glucose. It is noteworthy that the simultaneous expression of both an active PFK for glycolysis and an active FBPase for gluconeogenesis in the same glycosomes may generate a cycle leading to depletion of the glycosomal ATP. To prevent this potential futile cycle, the PFK and FBPase activities need to be controlled in BSF, for instance by reciprocal inhibition of the two enzymes by allosteric control and/or by post-translational modification. The absence of glucose in the insect vector midgut between bloodmeals may be the driving force of such an adaptation. However, it cannot be ruled out that FBPase is also required for BSF in a still unknown environmental niche. To our surprise, FBPase is ~8-fold more expressed in BSF compared to PCF (Fig 6A), which further strengthens its important role in BSF, presumably to feed gluconeogenesis.

A long-term growth of BSF cells in CMM_Glyc/GlcNAc induces an up- or down-regulation of 2.7% of the BSF proteome by at least 2-fold compared to CMM_Glc. This is far lower than the variations observed when comparing procyclic to slender BSF and slender BSF to stumpy BSF, showing 48% and 44% of at least 2-fold changes, respectively [42]. Among these long-term adaptations, glycerol metabolism is stimulated while glucose catabolism is reduced. Indeed, the rate of glycerol consumption, the oxygen consumption from glycerol metabolism and the excretion of end products from glycerol metabolism were increased by 11% (Fig 2C and 2D, Table 1), 22.5% (Fig 4A) and 19.2% (Fig 3), respectively, while the glucose consumption, the oxygen consumption from glucose metabolism and the excretion of end products from glucose metabolism were decreased by 15% (Fig 2C and 2D, Table 1), 8.2% (Fig 4A) and 17.9% (Fig 3), respectively. Interestingly, the increase of glycerol metabolism is correlated with the 1.8- and 1.5-fold up-regulation in the mitochondrial TAO and FAD-GPDH expressions, respectively. These enzymes are involved in the maintenance of the glycosomal redox balance through the glycerol phosphate shuttle and the mitochondrial oxidative capacity. This observation strongly suggests that the mitochondrial oxidative capacity is controlling the metabolic flow of the glycerol metabolism. It was previously reported that the expression of TAO can be modulated in response to stimuli and consequently may play a role in controlling the electron flow towards H_2_O production from O_2_. Indeed, BSF trypanosomes showed a ~2-fold increase of TAO expression in response to a continuous stress with ascofuranone, ultimately leading to trypanosome death [43]. Similarly, 12 h of incubation with 2 μM antimycin A or 50 μM H_2_O_2_, induced 1.6- and 2.1-fold increases of TAO activity, respectively, which was correlated to equivalent increases in TAO expressions [44]. Incidentally, the BSF proliferation in CMM_Glyc/GlcNAc is reduced, as compared to CMM_Glc, probably as a consequence of the limited TAO activity that may be responsible for the suboptimal capacity of oxygen consumption observed when glycerol is the only carbon source available. This reinforces our hypothesis that the mitochondrial oxidative capacity controls the glycerol metabolism.

Surprisingly, the first catalytic step of the glycerol metabolism (GK) is strongly down-regulated after the cell adaptation to CMM_Glyc/GlcNAc, with 4-fold and 7.8-fold reductions of the GK protein and activity, respectively, although the overall glycerol metabolism is concomitantly increased by ~20%. These counterintuitive data suggest that BSF present a large excess of GK activity that is not required to feed the central carbon metabolism from glycerol consumption and that is actually even detrimental to the parasite proliferation in glycerol-rich conditions. Although the reasons for this observed GK down-regulation are currently unknown, one may consider that the possible accumulation of glycerol 3-phosphate, the product of the GK enzymatic reaction, may affect the efficiency of downstream reactions. Indeed, it has previously been reported that incubation of the procyclic trypanosomes with glycerol induced a ~50-fold increase of intracellular amounts of glycerol 3-phosphate as compared to an incubation with glucose; this increase affecting the hexokinase activity [45].

Down-regulation of GK expression in CMM_Glyc/GlcNAc also suggests that BSF grown in glucose-rich conditions require the maintenance of this large excess of GK activity. In anaerobiosis, BSF produce equivalent amounts of pyruvate and glycerol from glucose breakdown and consequently need the GK activity to maintain the glycosomal redox balance required to sustain the glycolytic flow [21]. Interestingly, *in vitro* and *in vivo* studies have shown that the glycerol kinase activity considerably reduced the lethal effect of TAO inhibitors, which mimics anaerobiosis [46, 47]. This highlights the essential role of the GK for BSF during anaerobiosis. However, the relatively high oxygen tension in venous and arterial blood (pO_2_ in the range of 40 mmHg and 100 mmHg, respectively) is ~5-fold higher than the minimal oxygen tension required for the BSF to produced only pyruvate from glucose metabolism, which suggests that the BSF anaerobic pathway appears to be almost completely inoperative in the mammalian intravascular compartment [48]. However, it cannot be ruled out that BSF may encounter lower oxygen levels during their journey in mammalian tissues that would require GK expression for survival.

The association between trypanosomes and adipocytes, in mouse adipose tissues [10] as well as in the mouse skin [8], suggests that this interaction may be beneficial for the parasite by providing specific nutrients differing in nature and/or amounts from those available in the blood circulation. It has been proposed that fatty acids provided by the catabolism of triglycerides that are abundantly present in adipocytes may feed BSF ATP production through ß-oxidation [10]. Alternatively, one may consider that the glycerol also produced from triglyceride or phospholipid breakdown could be used as carbon source by BSF. Indeed, glycerol concentration is 5 to 20-fold higher in the interstitial fluids and adipose tissue than in plasma, as a result of local lipolysis [49, 50]. In addition, large amounts of glycerol can be produced by adipocytes from glucose and fructose breakdown through lipolysis-independent processes, in especially during hyperglycemia episodes [51, 52]. Incidentally, *T*. *brucei* infection in mice produced a condition resembling type 2 diabetes with decreased peripheral glucose utilization and increased glycemia during the first days of the infection [53, 54]. Consequently, one may consider that the ability of BSF trypanosomes to efficiently metabolise glycerol may favour the early colonisation of the adipose tissues. This adaptability could also be important for the maintenance of skin-dwelling parasites, possibly in the vicinity of dermal adipocytes [8], a prominent biological feature of the parasites for their effective transmission to tsetses [9]. Close interactions between pathogens and adipose tissues have already been described in the past [55]. For example, *Coxiella burnetti*, an obligate intracellular bacterium causing Q-fever, has recently been found inside mice adipocytes, that have consequently been assumed to serve as reservoir during the bacteria latency phase [56]. Incidentally, for its essential cell wall biosynthesis, this bacterium shows a preference for glycerol [57], which could be provided by adipocytes.

Overall, these results strengthen the newly illustrated paradigm that BSF trypanosomes are probably much more well adapted than previously admitted to the glucose-free/glycerol-rich conditions. No glucose-free environment has been described in mammals so far and the interstitial fluids contain glucose in the millimolar range, yet at a lower concentration compared to plasma. However, the high glycerol concentration in tissues as compared to that in plasma, particularly in the adipose tissues, suggests that parasites might encounter *in vivo* conditions compatible with the use of glycerol as a major carbon source. In addition, the resulting *in vivo* glycerol gradient between the intra and extravascular compartments would influence the parasite tropism to particular tissues via specific sensing pathways, such as the social motility phenomenon described in procyclic trypanosomes in the tsetse midgut [58]. Although the exact role of glycerol metabolism in BSF trypanosomes *in vivo* is not understood yet, our data open novel avenues for developing new diagnostic tools and/or treatments based on unexplored molecular targets.

## Materials and methods

### Trypanosomes and cell cultures

The bloodstream form of *T*. *brucei* 427 90–13 (TetR-HYG T7RNAPOL-NEO) [59], a 427 221a line (MiTat 1.2) designed for the conditional expression of genes was cultured at 37°C in IMDM (Iscove's Modified Dulbecco's Medium, Life Technologies) supplemented with 10% (v/v) heat-inactivated fetal calf serum (FCS), 0.25 mM ß-mercaptoethanol, 36 mM NaHCO_3_, 1 mM hypoxanthine, 0.16 mM thymidine, 1 mM sodium pyruvate, 0.05 mM bathocuprone and 2 mM L-cysteine [60]. The Creek’s minimal medium (CMM) was prepared as described before [25, 26] without glucose but with the addition of all amino acids at 0.1 mM. Cell culture grade (or high purity) components were purchased from Sigma-Aldrich. One-milliliter cultures were maintained in 24 wells plates at 37°C with 5% CO_2_. Cultures were grown to a maximum density of 3 x 10^6^ cells ml^-1^ and sub-cultured by 100- or 1,000-fold dilution every 2 or 3 days, respectively. Glucose-depleted CMM (CMM_Glyc/GlcNAc) was prepared by replacing glucose (10 mM) with 10 mM glycerol and adding 50 mM *N*-acetyl-D-glucosamine (GlcNAc), a non-metabolised glucose analogue inhibiting glucose transport, to prevent import of FCS-derived glucose (0.5 mM). The procyclic form of *T*. *brucei* EATRO1125.T7T (TetR-HYG T7RNAPOL-NEO) was cultivated at 27°C in the presence of 5% CO_2_ in SDM79 medium containing 10% (v/v) heat-inactivated fetal calf serum and 3.5 mg ml^-1^ hemin [61] or in a glucose-depleted medium derived from SDM79, called SDM79-GlcFree. This SDM79-GlcFree medium consists of a glucose-depleted SDM79 medium, containing 20% (v/v) heat-inactivated fetal calf serum, in which parental cells were cultured during 72 hours in order to consume the glucose coming from the serum and then diluted with the same volume of glucose-depleted SDM79 medium without serum to finally obtain SDM79-GlcFree. Glucose depletion was verified by NMR spectrometry analyses (with a detection threshold ≤ 1μM) and to prevent import of residual glucose 50 mM GlcNAc were added in the medium. Cell counts were obtained with a Guava EasyCyte Flow Cytometer (Merck Millipore).

### Inhibition of GK gene expression by RNAi

RNAi-mediated inhibition of expression of the *GK* genes (Tb927.9.12550-Tb927.9.12630) was performed in the 427 90–13 BSF by expression of stem-loop “sense-antisense” RNA molecules of the targeted sequences [62, 63] using the pLew100 expression vector, that contains the phleomycin resistance gene (kindly provided by E. Wirtz and G. Cross) [59]. To do so, a 617-bp fragment of the *GK* gene (from position 460 to 1077) was introduced in the pLew100 vector to produce the pLew-GK-SAS plasmid. Briefly, a PCR-amplified 617-bp fragment, containing the antisense GK sequence with restriction sites added to the primers, was inserted into the HindIII and BamHI restriction sites of the pLew100 plasmid. The separate 615-bp PCR-amplified fragment containing the sense GK sequence was then inserted upstream of the antisense sequence, using HindIII and XhoI restriction sites (XhoI was introduced at the 3'-extremity of the antisense PCR fragment). The resulting plasmid pLew100-GK-SAS contains a sense and antisense version of the targeted gene fragment, separated by a 89-bp fragment, under the control of a PARP promoter linked to a prokaryotic tetracycline operator. The ^*RNAi*^GK mutant was generated by transfecting the 427 90–13 parental cell line with the NotI-linearized pLew-GK-SAS plasmid, followed by selection in glucose-rich IMDM medium containing hygromycin (5 μg ml^-1^), neomycin (2.5 μg ml^-1^) and phleomycin (2.5 μg ml^-1^). Aliquots were frozen in liquid nitrogen as soon as possible after clone selection to provide stocks of each line. Induction of RNAi cell lines was performed by addition of 10 μg ml^-1^ tetracycline.

### Western blot analyses

Total protein extracts of *T*. *brucei* BSF cell lines harvested at a density of 10^6^ cells ml^-1^ (5 x 10^6^ cells per lane) were separated by SDS-PAGE (10%) and immunoblotted on TransBlot Turbo Midi-size PVDF Membranes (Bio-Rad) [64]. Immunodetection was performed as described [64, 65] using as primary antibodies, the rabbit anti-GK antibody (1:5,000, gift from P. Michels, Edinburgh, UK), the rabbit anti-hsp60 antibody (1:10,000) [66], the rabbit anti-ISG75 antibody (1:1,000, gift from P. Overath, Tubingen, Germany) [67], the rabbit anti-FBPase antibody (1:1,000, gift from P. Michels, Edinburgh, UK) and the mouse monoclonal anti-TAO antibody (7D3, 1:100, gift from M. Chaudhuri, Nashville, TN, USA) [68]. Anti-rabbit or anti-mouse antibodies conjugated to the horseradish peroxidase (Bio-Rad, 1:5,000 dilution) were used as secondary antibody. Revelation was performed using the Clarity Western ECL Substrate as described by the manufacturer (Bio-Rad). Images were acquired and analysed with the ImageQuant LAS 4000 luminescent image analyser (GE Healthcare).

### Determination of glucose and glycerol consumption

To determine the rate of glucose and glycerol consumption, BSF cells harvested at a density of 10^6^ cells ml^-1^ were grown in 10 ml of CMM_Glc, CMM_Glyc/GlcNAc or CMM_Glc/Glyc containing 5 mM of glucose and/or 5 mM of glycerol (inoculated at 2 x 10^6^ cells ml^-1^). Aliquots of growth medium (200 μl) were collected periodically during the 12 h of incubation at 37°C. The quantity of glucose and glycerol present in the medium was determined using the “Glucose GOD-PAP” kit (Biolabo SA) and the “Glycerol assay kit” (Sigma-Aldrich), respectively. The amount of carbon source consumed at a given time of incubation (Tx) was calculated by subtracting the remaining amounts in the spent medium at Tx from the initial amounts at T0. Then, the rate of glucose and glycerol consumed per h and per 10^8^ parasites was calculated from the equation of the linear curve deduced from plotting carbon source consumption as a function of time of incubation. Importantly, we controlled that 100% of the cells remained alive and motile at the end of the 12 h of incubation.

### Enzymatic activities

For enzymatic activities, BSF cells harvested at a density of 10^6^ cells ml^-1^ were washed in PBS (10 min, RT, 900 g), resuspended in assay buffer and after addition of “Complete EDTA-Free” protease-inhibitor cocktail (Roche) lysed by sonication (Bioruptor, Diagenode; high intensity, 5 cycles, 30sec/30sec on/off). Debris were spun down (1 min, RT, 16,000 g) and the supernatants were used for protein determination with the Pierce protein assay in a FLUOstar Omega plate reader at 660 nm. For higher throughput and smaller assay volumes all activity measurements were performed in a 96-well format with a FLUOstar Optima including an automated injection system. The baseline reactions were measured for 2 min and the reactions were started by injection of the specific substrate (glucose or glycerol) for each enzyme. The decrease/increase in absorbance at 350 nm was followed for 3–5 min. The rate was determined from the linear part of the progress curve and from this the specific activity was calculated. The buffer for glycerol kinase determination contains 100 mM triethanolamine pH 7.6, 2.5 mM MgSO_4_, 10 mM KCl, 0.6 mM ATP, 2 mM phosphoenolpyruvate, 0.6 mM NADH, ~1U lactate dehydrogenase, ~1U pyruvate kinase and 10 mM of glycerol (injected substrate). The buffer for hexokinase measurements contains 100 mM triethanolamine pH 7.6, 10 mM MgCl_2_, 0.6 mM ATP, 0.6 mM NADP^+^, ~1U glucose-6-phosphate dehydrogenase and 10 mM of glucose (injected substrate).

### Measurement of oxygen consumption

A total of 10^7^ cells were collected at the end of the exponential phase growth (10^6^ cells ml^-1^), centrifuged at 800 g for 5 min and resuspended in 2.3 ml of CMM_Glc or CMM_Glyc without FCS. Oxygen consumption rate was assessed at 28°C using 10^7^ cells per Oroboros O2K oxygraph chamber. The endogenous respiration of cells incubated in CMM_Glc or CMM_Glyc was recorded during steady state flux over several minutes. The SHAM sensitive respiration was then systematically determined by adding 4 mM SHAM, or from 1 nM to 1mM for EC50 determinations.

### NMR spectrometry experiments

BSF trypanosomes (10^7^ cells, ~0.1 mg of protein) were harvested at a density of 10^6^ cells ml^-1^ by centrifugation at 1,400 g for 10 min, washed once with phosphate-buffered saline (PBS) containing 1 mM of the carbon source and incubated for 1.5 h at 37°C in 1 ml of incubation buffer (PBS supplemented with 5 g l^-1^ NaHCO_3_, pH 7.4), with [U-^13^C]-glucose or glucose (4 mM) in the presence or the absence of glycerol (4 mM). The integrity of the cells during the incubation was checked by microscopic observation. 50 μl of maleate (10 mM) were added as internal reference to a 500 μl aliquot of the collected supernatant and proton NMR (^1^H-NMR) spectra were performed at 500.19MHz on a Bruker Avance III 500 HD spectrometer equipped with a 5 mm cryoprobe Prodigy. Measurements were recorded at 25°. Acquisition conditions were as follows: 90° flip angle, 5,000 Hz spectral width, 32 K memory size, and 9.3 sec total recycle time. Measurements were performed with 64 scans for a total time close to 10 min 30 sec. Resonances of the obtained spectra were integrated and metabolites concentrations were calculated using the ERETIC2 NMR quantification Bruker program.

Two-tailed Student’s t-tests for unpaired samples were performed to analyse the effects of adaptation to CMM_Glyc/GlcNAc on the total excreted products in the 427 90–13 cell line. The comparisons were performed between parasites grown in CMM_Glyc/GlcNAc *versus* CMM_Glc both incubated with the same carbon sources (glucose, glycerol or glucose/glycerol). Similar statistical analyses were done for the ^*RNAi*^GK cell line to compare the tetracycline-induced *versus* non-induced parasites incubated in glucose or glycerol.

### Mass spectrometry analyses of intracellular metabolites

For analysis of [^13^C]-incorporation into intracellular metabolites, BSF cells grown in IMDM at a density of 10^6^ cells ml^-1^ were washed once with PBS containing 0.1 mM glycerol and 2 x 10^7^ cells were resuspended in 5 ml incubation solution (PBS containing 2 mM [U-^13^C]-glycerol with or without 2 mM glucose). The cells were incubated for 1 h at 37°C, then transferred in a 50 ml plastic tube to cool down the medium in a dry ice/ethanol bath (20 sec), followed by centrifugation at 4°C at 1,400 g for 5 min. The cell pellet was then frozen in liquid nitrogen for mass spectrometry analysis, as described before [25, 26]. The extraction of intracellular metabolites was carried out by adding 2 ml of a cold extraction solution (acetonitrile/methanol/water, 4:4:2 v/v at -20°C, containing 0.1% formic acid v/v). The extracts were briefly vortexed (**~**2 sec) and immediately incubated at -20°C for 20 min. After evaporation, the dried extracts were resuspended in 150 μl ultrapure water and centrifuged to remove cell residues prior to analysis. The analyses of metabolites were carried out on liquid anion exchange chromatography Dionex ICS-5000+ Reagent-Free HPIC (Thermo Fisher Scientific™, Sunnyvale, CA, USA) system coupled to QExactive Plus high resolution mass spectrometer (Thermo Fisher Scientific, Waltham, MA). Central metabolites were separated within 48 min, using linear gradient elution of KOH applied to an IonPac AS11 column (250 x 2 mm, Dionex) equipped with an AG11 guard column (50 x 2 mm, Dionex) at a flow rate of 0.35 ml min^-1^. The column and autosampler temperature were 30°C and 4°C respectively. Injected sample volume was 15 μl. Mass detection was carried out in a negative electrospray ionization (ESI) mode. The settings of the mass spectrometer were as follows: spray voltage 2.75 kV, capillary and desolvatation temperature were 325°C and 380°C respectively, maximum injection time 100 ms. Nitrogen was used as sheath gas (pressure 50 units) and auxiliary gas (pressure 5 units). The automatic gain control (AGC) was set at 1e6 for full scan mode with a mass resolution of 140,000. Identification of ^13^C carbon isotopologue distribution relied upon matching accurate masses from FTMS (mass tolerance of 5 ppm) and retention time using TraceFinder 3.2 software. Measurement of alanine ^13^C incorporation was performed following the method described in Heuillet *et al*. [69] To obtain ^13^C-labelling patterns (^13^C isotopologues), isotopic clusters were corrected for the natural abundance of isotopes of all elements and for isotopic purity of the tracer, using the in-house software IsoCor (available at MetaSys) [70].

The metabolites under examination included alanine, fructose 1,6-bisphosphate (F1,6BP), fructose 6-phosphate (F6P), fumarate, glucose 6-phosphate (G6P), glycerol 3-phosphate (Gly3P), malate, mannose 6-phosphate (M6P), phosphoenolpyruvate (PEP), 6-phosphogluconate (6-PG), 2- and 3-phosphoglycerate (2/3PG), ribose 5-phosphate, ribulose 5-phosphate, xylulose 5-phosphate (considered together as pentose 5-phosphate, Pentose5P) and succinate.

### Sample preparation for proteomic analysis

This analysis was performed by the proteomics core facility at University of Bordeaux (https://proteome.cgfb.u-bordeaux.fr/en). The steps of sample preparation and protein digestion were performed as previously described [71].

### NanoLC-MS/MS analysis

Online nanoLC-MS/MS analyses were performed using an Ultimate 3000 RSLC Nano-UPHLC system (Thermo Scientific, USA) coupled to a nanospray Q Exactive hybrid quadrupole-Orbitrap mass spectrometer (Thermo Scientific, USA). 500 ng of each peptide extract was loaded on a 300 μm ID x 5 mm PepMap C_18_ precolumn (Thermo Scientific, USA) at a flow rate of 10 μl min^-1^. After a 3 min desalting step, peptides were separated on a 75 μm ID x 25 cm C_18_ Acclaim PepMap RSLC column (Thermo Scientific, USA) with a 4–40% linear gradient of solvent B (0.1% formic acid in 80% ACN) in 108 min. The separation flow rate was set at 300 nl min^-1^. The mass spectrometer operated in positive ion mode at a 1.8 kV needle voltage. Data was acquired using Xcalibur 3.1 software in a data-dependent mode. MS scans (m/z 300–1600) were recorded at a resolution of R = 70,000 (m/z 200) and an AGC target of 3 × 10^6^ ions collected within 100 ms. Dynamic exclusion was set to 30 s and top 12 ions were selected from fragmentation in HCD mode. MS/MS scans with a target value of 10^5^ ions were collected with a maximum fill time of 100 ms and a resolution of R = 17,500. Additionally, only +2 and +3 charged ions were selected for fragmentation. Other settings were as follows: no sheath and no auxiliary gas flow, heated capillary temperature, 200°C; normalized HCD collision energy of 27 eV and an isolation width of 2 m/z.

### Database search and results processing

For protein identification, Sequest HT and Mascot 2.4 algorithms through Proteome Discoverer 1.4 Software (Thermo Fisher Scientific Inc.) were used for protein identification in batch mode by searching against a *T*. *brucei* protein database (9,594 entries, TriTrypDB-33_TbruceiTREU927, release 33). This database was downloaded from http://tritrypdb.org website. Two missed enzyme cleavages were allowed. Mass tolerances in MS and MS/MS were set to 10 ppm and 0.02 Da. Oxidation of methionine, acetylation of lysine and deamidation of asparagine and glutamine were searched as dynamic modifications. Carbamidomethylation on cysteine was searched as static modification. Peptide validation was performed using Percolator algorithm and only “high confidence” peptides were retained corresponding to a 1% False Discovery Rate (FDR) at peptide level [72].

### Label-Free quantitative data analysis

Raw LC-MS/MS data were imported in Progenesis QI (version 2.0; Nonlinear Dynamics, a Waters Company) for feature detection, alignment, and quantification. All sample features were aligned according to retention times by manually inserting up to fifty landmarks followed by automatic alignment to maximally overlay all the two-dimensional (m/z and retention time) feature maps. Singly charged ions and ions with higher charge states than six were excluded from analysis. All remaining features were used to calculate a normalization factor for each sample that corrects for experimental variation. Peptide identifications (with FDR<1%) were imported into Progenesis. Only non-conflicting features and unique peptides were considered for calculation of quantification at protein level. A minimum of three peptides matched to a protein was used as the criteria for identification as a differentially expressed protein. Univariate one-way analysis of variance (ANOVA) was performed within Progenesis to calculate the protein p-value according to the sum of the normalized abundances across all runs. Only protein with a p-value cutoffs ≤ 0.05 were validated. The mass spectrometry proteomics data have been deposited to the ProteomeXchange Consortium via the PRIDE [73] partner repository with the dataset identifier PXD009763.

## Supporting information

S1 Fig NMR analysis of end products excreted from the metabolisms of glucose (panel A) and glycerol (panel B) by the tetracycline-induced (.i) and non-induced (.ni) ^*RNAi*^GK cell line.
(PDF)

S1 Table Proteins up- or down-regulated at least by 2-fold in BSF adapted to CMM_Glyc/GlcNAc. (XLS)

S2 Table Proteome comparison between BSF adapted to CMM_Glc and CMM_Glyc/GlcNAc. (XLS)

S3 Table Comparison of the expression of proteins involved in glucose and glycerol metabolism between BSF adapted to CMM_Glc and CMM_Glyc/GlcNAc. (XLS)
