# Supplementary material for: Glycerol supports growth of the Trypanosoma brucei bloodstream forms in the absence of glucose: Analysis of metabolic adaptations on glycerol-rich conditions
Source: PLoS Pathog. 2018 Nov 1;14(11):e1007412. doi: 10.1371/journal.ppat.1007412 (PMC6245841; doi:10.1371/journal.ppat.1007412)

S1 Fig. NMR analysis of end products excreted from the metabolisms of glucose (panel A) and glycerol (panel B) of the tetracycline-induced (.i) and non-induced (.ni) ^{RNAi}GK cell line. The excreted end products are pyru"vate (Pyr), alanine (Ala), acetate (Ace) and succinate (Suc) and only a part of each spectrum ranging from 1.2 ppm to 4.0 ppm is shown.

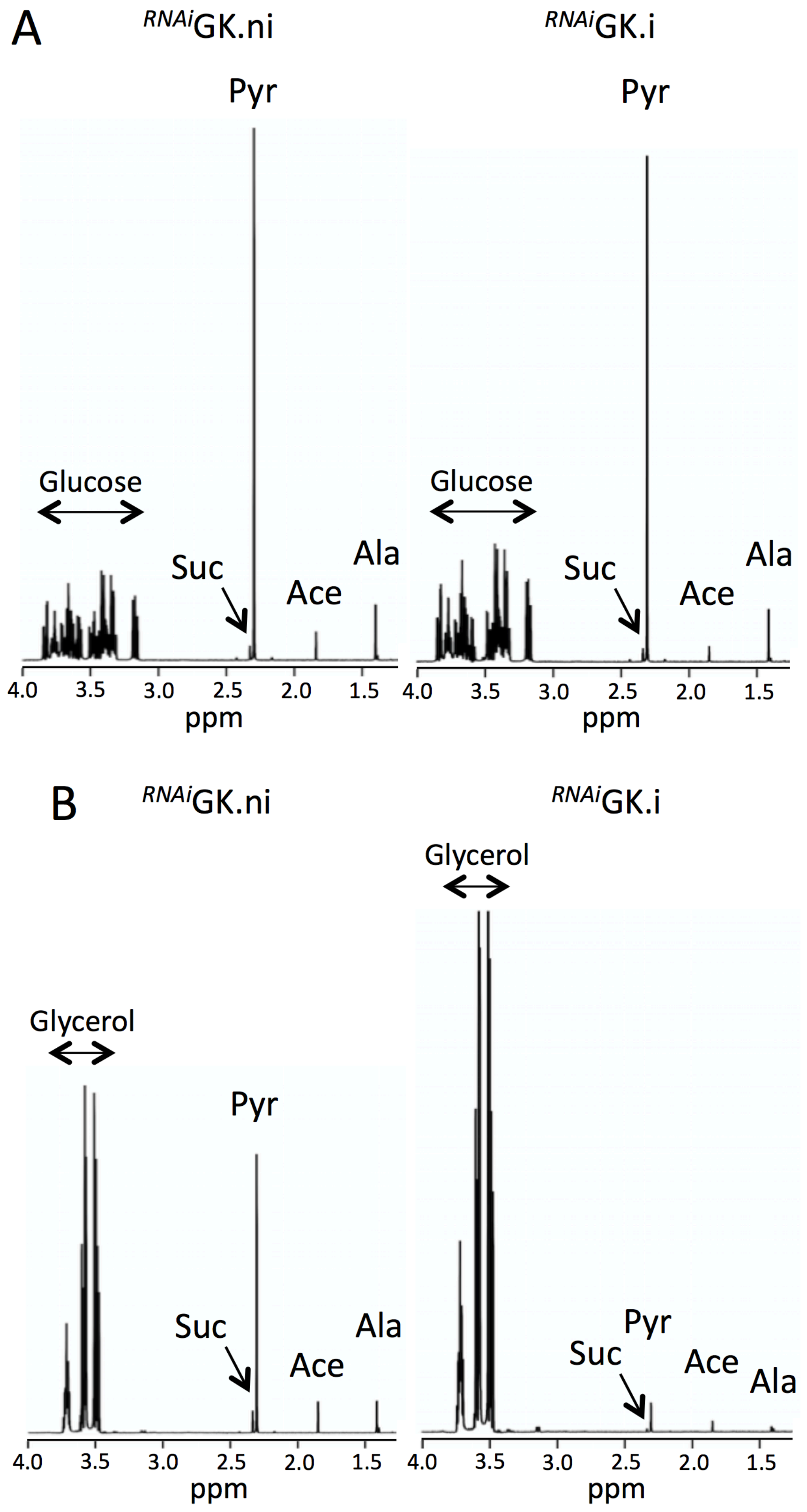

Supplement: S1 Fig — NMR analysis of end products excreted from the metabolisms of glucose (panel A) and glycerol (panel B) by the tetracycline-induced (.i) and non-induced (.ni) RNAiGK cell line. (PDF) [file ppat.1007412.s001.pdf]
